# Active Transiency: A Novel Approach to Expedite Degradation in Transient Electronics

**DOI:** 10.3390/ma13071514

**Published:** 2020-03-26

**Authors:** Reihaneh Jamshidi, Yuanfen Chen, Reza Montazami

**Affiliations:** 1 Department of Mechanical Engineering, University of Hartford, West Hartford, CT 06117, USA; 2 College of Mechanical Engineering, Guangxi University, Nanning 530004, China; 20180013@gxu.edu.cn; 3 Department of Mechanical Engineering, Iowa State University, Ames, IA 50011, USA; reza@iastate.edu

**Keywords:** transient electronics, polymer composites, active transiency

## Abstract

Transient materials/electronics is an emerging class of technology concerned with materials and devices that are designed to operate over a pre-defined period of time, then undergo controlled degradation when exposed to stimuli. Degradation/transiency rate in solvent-triggered devices is strongly dependent on the chemical composition of the constituents, as well as their interactions with the solvent upon exposure. Such interactions are typically slow, passive, and diffusion-driven. In this study, we are introducing and exploring the integration of gas-forming reactions into transient materials/electronics to achieve expedited and active transiency. The integration of more complex chemical reaction paths to transiency not only expedites the dissolution mechanism but also maintains the pre-transiency stability of the system while under operation. A proof-of-concept transient electronic device, utilizing sodium-bicarbonate/citric-acid pair as gas-forming agents, is demonstrated and studied vs. control devices in the absence of gas-forming agents. While exhibiting enhanced transiency behavior, substrates with gas-forming agents also demonstrated sufficient mechanical properties and physical stability to be used as platforms for electronics.

## 1. Introduction

Transient electronics is an emerging technology with paramount potentials in a wide range of applications such as biomedical devices [1,2,3], environmental sensors, and hardware security [4,5]. Examples of transient electronic devices include transistors [1], mechanical energy harvesters [6], energy storage devices [7,8] and functional circuits for radio frequency (RF) transmission [9,10].

Early works on transient materials and electronics can be divided into three main categories: (i) electronics on bioresorbable substrates [11], (ii) partially transient electronics with specific degradable components [2], and (iii) fully dissolvable electronics [12]. Most of the previous studies in this field have focused on dissolution based transiency [1,12,13,14,15,16,17,18]. Thermally and optically triggered transiency have also been studied [19,20,21], yet to a lesser extent. In two separate studies, Park et al. and Hernandez et al. have reported thermally [19] and optically [21] triggered systems where acid generation derives the disintegration of a system. In a study by Pal et al. enzymatic degradation of fully organic biosensors, based on poly(3,4-ethylenedioxythiophene):poly (styrene sulfonate) (PEDOT:PSS) conductive ink and silk substrate, has been investigated and reported [22].

Several groups have extensively investigated applicable materials in the field of transient electronics and their degradation behaviors [1,4,15,18,23,24,25,26,27,28]. Materials applicable as substrates in transient electronics have included thermoplastic polymers such as poly(L-lactide-co-glycolide) (PLGA) [2] and poly(vinyl alcohol) (PVA) [14], elastomers such as poly(glycerol-co-sebacate) (PGS) [29] and poly(1,8-octanediol-cocitrate) (POC) [4], and even natural materials such as chitosan and rice paper [30,31].

The desired degradation time for transient materials is a critical parameter that is highly dependent on the application. In the case of hardware security, the appropriate degradation time is expected to be significantly shorter than that of biomedical devices. Rogers et al. have reported materials and fabrication techniques that facilitate transiency within few days [12,32], mainly applicable to biomedical devices. In a previous study, we reported substrates for transient electronics with a programmable degradation rate to accommodate controlled transiency within minutes [14].

Degradation time in dissolution based systems is strongly dependent on the chemical composition of the constituents, as well as their interactions with the solvent. Such interactions are typically slow, passive, and diffusion-driven. Here, we are introducing and exploring a new transiency approach by designing transient materials to undergo selective, active, and fast chemical reactions with their environment (solvent) and generate physical force that could be utilized in hybrid transiency and disintegration/redispersion of inherently non-transient components. This is achieved by the integration of gas-forming agents into the transient materials to achieve selective, expedited, and active transiency via gas-forming chemical reactions that are initiated in a controlled manner. The integration of more complex chemical reaction paths to transiency not only increases the selectivity of the process but also enhances pre-transiency chemical stability of the system while under operation.

**Figure 1:**
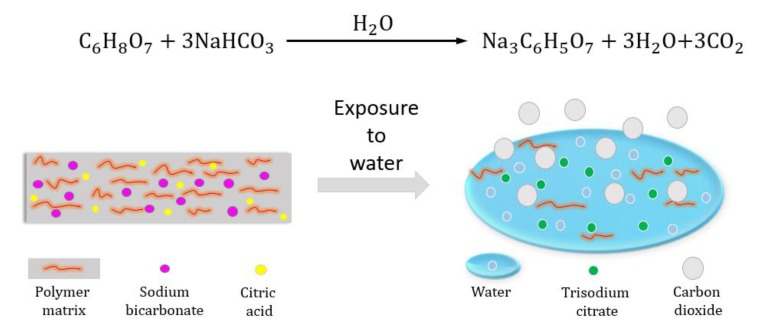
A structural model of active transiency by means of gas-forming reactions.

A proof-of-concept transient electronic device, with non-transient electronic components (silver ink) and a substrate that consists of a water-soluble polymer matrix (polyethylene oxide (PEO), PVA, and gelatin) doped with a pair of gas-forming agents (sodium-bicarbonate (NaHCO_3_)/citric-acid (C_6_H_8_O_7_)) are demonstrated and studied vs. control devices in the absence of gas-forming agents. The released gas, and the resultant force, are harnessed to expedite the transiency of the device (Figure 1). The control substrate is composed of a precursor PVA and Gelatin film, crushed into powder, and combined with PEO in ethanol solvent. PVA is selected for its biocompatibility and desirable physical and chemical properties. Gelatin is used as a means to increase the stiffness of precursor film due to the presence of triple helixes in its structure, and thus facilitate PVA-gelatin’s powder formation [14]. PEO was intended to enhance the flexibility of the substrate due to its low molecular weight [13].

Findings reported here could be extended to other material systems, consisting of different matrices and gas-forming agents, to enhance selectivity and limit transiency to more specific conditions such as pH and temperature, among others.

## 2. Materials and Methods

### 2.1. Materials

Poly(vinyl alcohol) (PVA) (Mowiol 10–98, M_W_: 61,000 g·mol^−1^, 98.0–98.8 mol% hydrolyzed), gelatin (gel strength ~175 g Bloom, Type A), poly(ethylene oxide) (PEO) (Mw 400,000), citric acid and sodium bicarbonate were purchased from Sigma-Aldrich (St. Louis, MO, USA). Conductive silver ink (Pelco, 187) was purchased from Ted Pella (Redding, CA, USA). Ethanol (90%) was purchased from BDH Chemicals (Randor, PA, USA).

### 2.2. Preparation of Polymer Films

To prepare polymer films’ precursor 1 g of PVA and 2 g of gelatin were added to 20 mL of deionized (DI) water (R ≥ 18.0 MΩ), and the solution was stirred at 90 °C for 2 h, then it was cast on plastic mold and dried at ambient conditions for 24 h. The film was then carefully peeled off from the mold and crushed into powder. For making the base film (control) 0.250 g of gelatin-PVA (GPVA) powder was added to 0.250 g of PEO and stirred with 1 mL of ethanol. To make samples of various citric acid and sodium bicarbonate concentrations, desired amounts of each were added to the mixture of PVA gelatin powder and PEO in ethanol. The mixtures were compacted under 3.125 MPa pressure for 1 min. The resultant film thickness was 0.16 mm. Presented in Table 1 is the chemical composition of each sample, along with the notation used in this study. The weight percentage of each sample is calculated over the weight of the ethanol solvent.

### 2.3. Electrically Conductive Patterns

Electrically conductive silver ink was diluted with acetone (1:1 volume ratio) and sprayed on the polymer membranes over a vinyl mask of the desired pattern with an open area of 25 mm^2^. Vinyl masks with desired circuit designs were fabricated using a vinyl cutter (US-Cutter, SC series, Seattle, WA, USA) with 25 µm planar resolution and 125 µm repetition accuracy. Film thickness was controlled by monitoring the electric conductivity of the sprayed pattern.

### 2.4. Transiency

#### 2.4.1. Substrates

The transiency of the polymer films was defined as normalized mass loss and determined as the ratio of the film’s mass before and after exposure to the trigger, DI water. The 1 × 3 cm^2^ pieces of polymer films were individually sandwiched in 3 × 4 cm^2^ aluminum-mesh containers. The mesh containers, containing polymer films, were submerged in DI water for 0, 10, 30, 60, 120 and 300 s; then dried in air for 24 h. The mass of the containers and the films were measured and recorded before and after exposure to calculate transiency (mass loss). All the experiments were repeated at least three times, and the results were averaged.

#### 2.4.2. Devices

The transiency of circuits was deduced from measuring the resistivity of circuits as a function of exposure time to DI water. Circuits were degraded in a petri dish filled with DI water; external electrodes were isolated from contact with the solution to prevent short-circuiting through the solution.

### 2.5. Mechanical Characterizations

The tensile properties of the polymer films were determined using a dynamic mechanical analyzer (DMA-1, Mettler Toledo, Columbus, OH, USA) loaded with tension clamps, at static mode. Static testing was performed on force-controlled mode for a range of 0–5 N at a rate of 0.1 Nmin^−1^. The elastic modulus (E) and yield stress (S_y_) were calculated from the resultant force and displacement data. All mechanical characterizations were carried under isothermal conditions. For strain-electric properties correlations under static load, clamps were insulated by tape to prevent short circuit when electrical measurements were taken.

### 2.6. Infrared Spectroscopy

Fourier transformed infrared (ATR-FTIR) spectroscopy (Frontier PerkinElmer, Waltham, MA, USA) was used for the chemical characterization of the samples. Four scans with a spectral resolution of 4 cm^−1^ were taken at room temperature for each sample.

### 2.7. Electrical Characterization

Electronic properties of circuits under stress were monitored and recorded on a potentiostat (VersaSTAT 4, Princeton Applied Research, Oak Ridge, TN, USA) using VersaStudio software (Version 2.00, AMETEK Scientific Instruments, Berwyn, PA, USA). Bias potential of 1 V was applied during the experiments, and resistance was measured and recorded by the software.

## 3. Results and Discussion

### 3.1. Transiency

#### 3.1.1. Substrates

**Figure 2:**
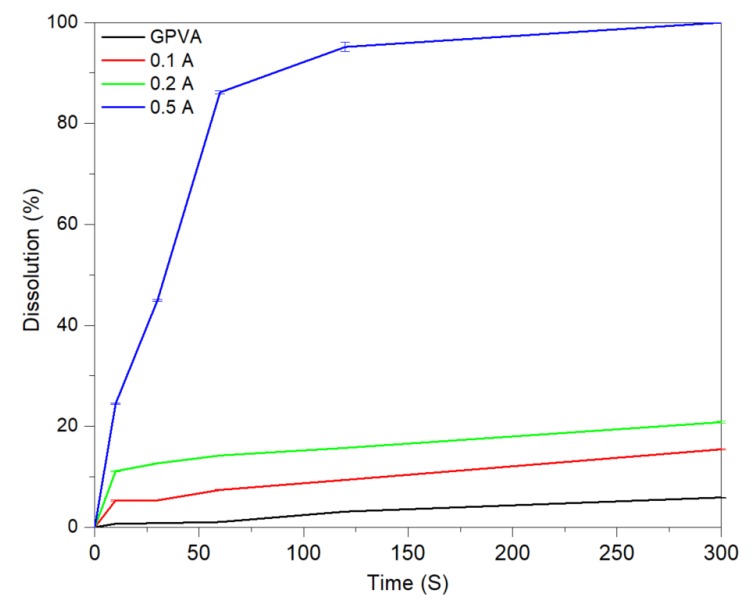
Time-dependence dissolution of substrates.

The composite films exhibited high environmental stability. Samples did not show any detectable signs of degradation or change in properties under ambient conditions (~25 °C, ~65% humidity) over a period of six months. Presented Table 1 is the chemical composition of the samples, and Figure 2 demonstrates the time-dependence dissolution (%) for samples of different compositions. The results indicate that solubility is highly dependent on the additive concentration, which determines the extent of reaction with water. In the presence of water, sodium bicarbonate and citric acid undergo a set of endothermic reactions that form sodium citrate, water, and carbon dioxide. The resultant carbon dioxide is released in the form of small bubbles, exerting mechanical force on the structure resulting in expedited hybrid transiency of the films.

**Figure 3:**
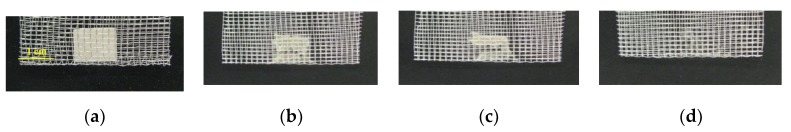
Sequential images of degradation of 0.5 A substrate. (**a**) 0 s; (**b**) 10 s; (**c**) 30 s; (**d**) 300 s.

In a previous study, we defined the transiency threshold as the time at which 63.3% of the original mass of the sample is lost [14]. Within 5 min of exposure to DI water, the GPVA-PEO, 0.1 A and 0.2 A samples lost approximately 6% to 20% of their original masses, respectively. The 0.5 A samples, however, reached transiency threshold within 40 s, which is desirable for applications where spontaneous dissolution is preferred. Figure 3 demonstrates the sequential degradation of a 0.5 A sample upon exposure to DI water, and its complete mass loss over 300 s.

#### 3.1.2. Devices

**Figure 4:**
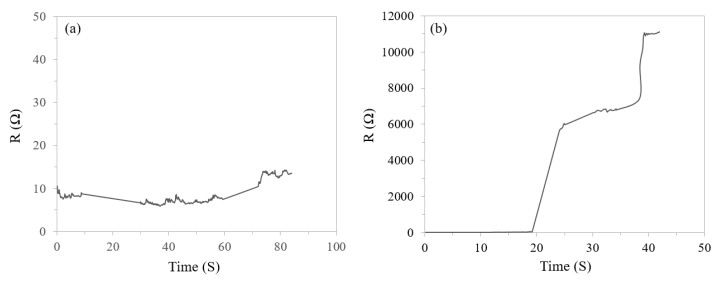

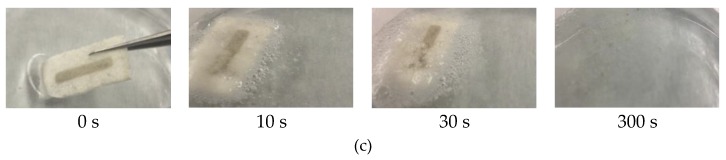
Transiency of a straight conductive path fabricated on (**a**) GPVA-PEO substrate and (**b**) 0.5 A substrate quantified from loss of electrical conductivity when exposed to DI water (1.5 % reading accuracy for resistance); (**c**) sequential images of gas formation and transiency of 0.5 A substrate with a straight conductive path.

The current study is aimed to investigate the effect of additives on the transiency of the whole system. For this purpose, identical patterns of the electrically conductive path were fabricated on the GPVA-PEO and 0.5 A substrates (a schematic of the fabrication process is presented and explained in our previous study) [10], and resistance was monitored and recorded as a function of exposure time to DI water. Presented in Figure 4 are the resistance curves for the GPVA-PEO and the 0.5 A samples with a conductive path. The GPVA-PEO sample demonstrates a minor overall resistance increase over 85 s (Figure 4a); this is in agreement with the slow dissolution rate of the control film. The 0.5 A sample (Figure 4b), however, initiates a significant resistance increase after 19 s, which indicates the spontaneous dissolution of the substrate as a result of undergoing gas-forming reactions. As observed in Figure 4b, the transiency of the 0.5 A sample slows down after 25 s; this is attributed to the lower concentration of the gas-forming agents remained in the system. Further reactions and dissolution of the substrate cause a lack of mechanical support for the circuit, resulting in instability of the device and very high resistance values after 85 s. Transiency experiments presented here suggest that the transiency rate of the substrate is dominating the mechanism of degradation and transiency of the whole device. Degradation and transiency rates of the bioelectronic devices can be tuned by controlling the transiency of the substrate through the addition of gas-forming agents. Figure 4c depicts sequential images of dissolution by means of gas formation for 0.5 A substrate with a straight conductive path.

### 3.2. Mechanical Characterizations

**Figure 5:**
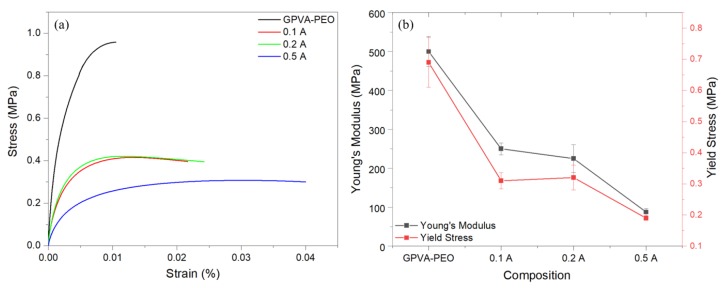
(**a**) characteristic stress-strain behavior of the substrates, (**b**) elastic modulus (E) and yield stress (S_y_) of the substrates.

The effect of chemical composition on the mechanical properties of samples was investigated. Figure 5a demonstrates characteristic stress-strain profiles of the samples as a function of gas-forming agent content. It is evident from the graphs that increasing the sodium bicarbonate and citric acid constituents increase the stretchability of the substrates. Demonstrated in Figure 5b are the elastic modulus (E) and yield stress (S_y_) for samples of different compositions. The addition of sodium bicarbonate and citric acid corresponds to a decrease in both elastic modulus and yield stress, and both values follow a similar trend. This behavior is anticipated from the low molecular weight of the sodium bicarbonate and citric acid (84 and 192 g·mol^−1^, respectively) compared to that of the constituent polymers. The addition of components with smaller molecular weights acts as a plasticizer, followed by a decrease in elastic modulus and yield stress. These observations are in agreement with those of a recent study on a similar composition, PVA/sodium-bicarbonate [8]. Increasing the additive content from 10% to 20% (corresponding to 0.1 A and 0.2 A samples) did not result in a significant change in the mechanical properties of the substrates; however, an indicative decrease in elastic modulus and yield stress were observed between the 0.2 A and 0.5 A samples. Such a decrease in elastic modulus may facilitate the use of these transient substrates for flexible and/or stretchable electronics.

**Figure 6:**
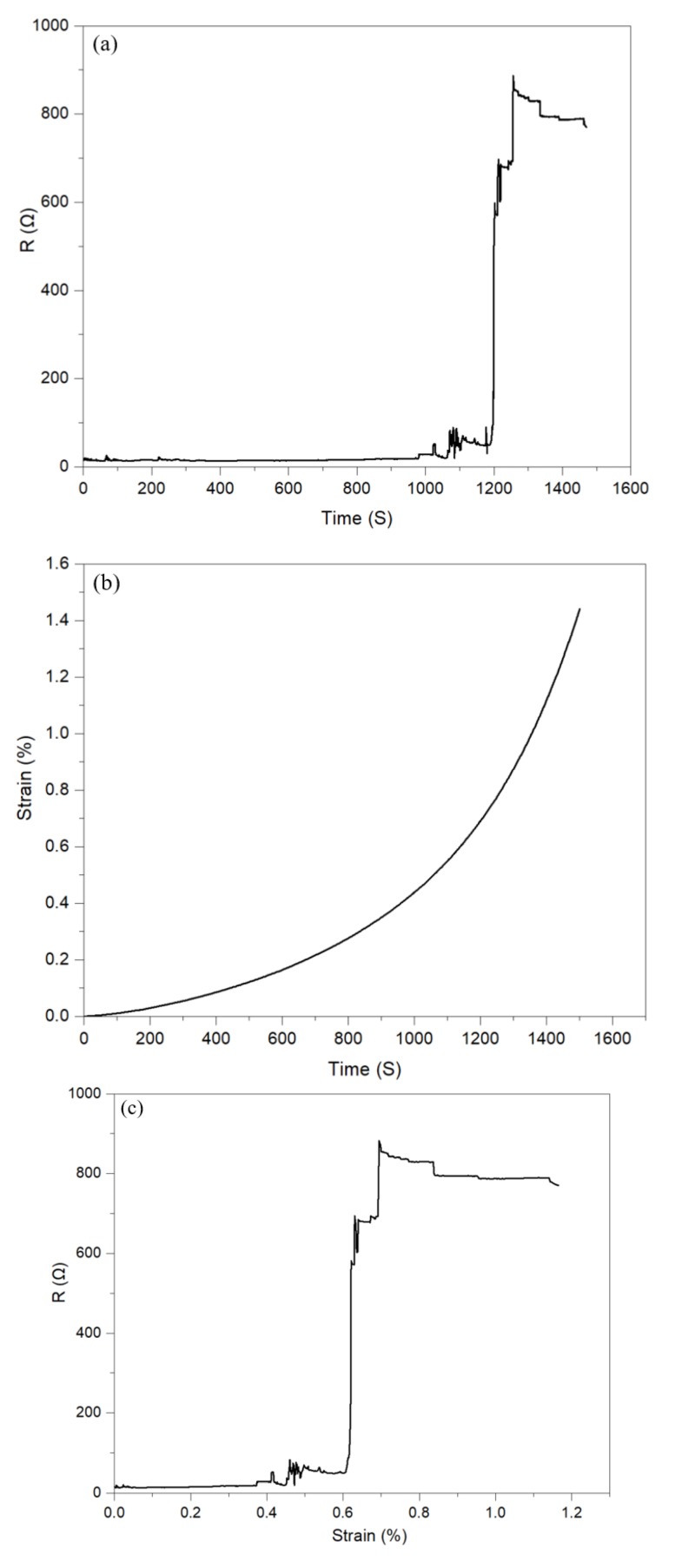
(**a**) Resistance over time upon the applied strain, (**b**) strain curve over time upon the applied force from 0–5 N, and (**c**) resistance over the applied strain (1.5 % reading accuracy for resistance).

Strain-electrical properties correlations under static load: resistance of a basic circuit consisting of a single electrode on the 0.5 A substrate was investigated in response to strain resulted from a force of 1–5 N increasing at 0.1 N·min^−1^ increments. As demonstrated in Figure 6a,b, the resistance and strain were monitored and recorded as a function of time, and resistance was reported as a function of strain in Figure 6c. Figure 6a demonstrates a linear increase in resistance up to 500 s and exhibits a non-linear second-order behavior beyond that point until the experiment is concluded at 1500 s. In Figure 6b, the resistance remained unchanged for up to 1000 s and increased uniformly up to 1200 s; then, an abrupt increase started at ca. 1200 s. The unchanged resistance values and the abrupt increase at 1200 s result from the low flexibility of the film and that of conductive silver ink. The narrow liner elastic region of the substrates is demonstrated in Figure 6b, and the inelastic behavior of the conductive ink patterns on PVA film was studied in our previous work [10]. The resistance vs. strain data presented in Figure 6c confirms unchanged resistance for up to 0.37% strain, followed by a uniform increase up to 0.60% strain, and an abrupt increase beyond that.

### 3.3. Chemical Characterization

**Figure 7:**
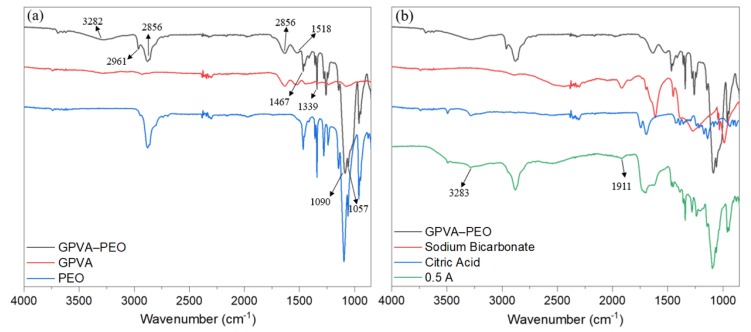
FTIR spectra of (**a**) GPVA-PEO substrate compared to GPVA and PEO (**b**) 0.5A substrate compared to GPVA-PEO, sodium bicarbonate, and citric acid.

FTIR spectroscopy was used to investigate the interactions within the polymers, and between the polymers and the gas-forming agents. Presented in Figure 7a is the FTIR spectra of a GPVA-PEO film, GPVA, and PEO. FTIR spectrum of GPVA film showed a broad peak at 3282 cm^−1^, which corresponds to the stretching vibrations of hydroxyl groups in PVA [14,33]. The peaks at 2961 and 2856 cm^−1^ in GPVA-PEO film are assigned to stretching of C-H groups in PEO. The band at 1650 cm^−1^ is a characteristic peak of gelatin and is assigned to amide I (C=O and C–N stretching vibration) [34]. The band at 1467 cm^−1^ was attributed to bending vibrations of C–H groups of PEO coupled with bending vibrations of O–H groups at 1339 cm^−1^ [14]. The peaks at 1090 and 1058 cm^−1^ are assigned to stretching of C–O group [35]. Figure 7b represents the FTIR spectra of GPVA-PEO and 0.5 A films, sodium bicarbonate, and citric acid.

The 0.5 A sample exhibited additional peaks to the GPVA-PEO. A shoulder appeared at 3283 cm^−1^, which was attributed to the stretching of the carboxylic acid O–H group in citric acid. The formation of the peak at 1911 cm^−1^ was assigned to the stretching of C=O group in sodium bicarbonate [33].

In general, the FTIR spectra did not identify the formation of chemical bonds at the interface of the polymers, or between the polymers and the gas-forming agent. This observation confirms that the gas-forming agents do not undergo any chemical reaction with the matrix materials and maintain their desired chemical properties to trigger transiency once needed.

## 4. Conclusions

The presented results demonstrate that controlled, expedited, and active transiency in a transient materials system is achieved by initiating gas-forming chemical reactions. Polymer composites consisting of different ratios of sodium bicarbonate and citric acid as gas-forming agents, integrated with a water-soluble polymer matrix (polyethylene oxide (PEO), PVA, and gelatin), were tested for their dissolution behavior as well as physical and chemical properties. While an expedited transiency was proved for samples containing the gas-forming additives, they also demonstrated sufficient mechanical properties and physical stability to be applied as substrates for transient electronics. It was also observed that the addition of these additives results in higher stretchability, yet lower yield stress for the composites. If subjected to mechanical strain greater than ca. 0.35%, the device presented here is vulnerable to malfunction due to the electrical variations as a function of strain. Therefore, the development of linear elastic substrates and conductive patterns appears to be necessary for flexible electronics applications. However, the present materials and method facilitate the spontaneous dissolution of the devices when required.

## Figures and Tables

**Table 1:** Sample notations and chemical compositions of the synthesized films.

Notation	GPVA (wt%)	PEO (wt%)	Sodium Bicarbonate (wt%)	Citric Acid (wt%)
GPVA-PEO	25	25	0	0
0.1 A	25	25	2.5	2.5
0.2 A	25	25	5	5
0.5 A	25	25	12.5	12.5
